#  miR-217 Is a Useful Diagnostic Biomarker and Regulates Human Podocyte Cells Apoptosis via Targeting TNFSF11 in Membranous Nephropathy

**DOI:** 10.1155/2017/2168767

**Published:** 2017-10-30

**Authors:** Jing Li, Bin Liu, Hen Xue, Qiao Qiao Zhou, Ling Peng

**Affiliations:** ^1^Department of General Medicine, Sichuan Cancer Hospital, Chengdu 610041, China; ^2^Department of Medical Oncology, Sichuan Cancer Hospital, Chengdu 610041, China; ^3^Department of Nephrology, Ya'an People's Hospital, Ya'an 625000, China

## Abstract

**Background:**

MicroRNAs have recently been verified as useful diagnostic biomarkers in various diseases. In this study, we investigated whether miR-217 is a useful diagnostic biomarker and the possible pathological mechanism of miR-217 in this disease.

**Methods:**

Patients with focal segmental glomerulosclerosis (FSGS), membranous nephropathy (MN), and diabetic nephropathy (DN) and control patients were enrolled in this study. The miR-217 inhibitor and mimics were transfected into human podocyte cells to investigate the pathological mechanism of miR-217 in this disease. Relevant indicators were detected and tested.

**Results:**

Compared with control patients, miR-217 was significantly downregulated and TNFSF11 was significantly upregulated in MN. Then, miR-217 had obvious separation between patients with MN and control patients, with an AUC of 0.941, a cutoff value of <750.0 copies/ul, and sensitivity and specificity of 88.9% and 75.9%. In addition, the TNFSF11 was confirmed to be the target gene of miR-217. Finally, in in vitro experiments, the upregulation of miR-217 could decrease the expression of TNFSF11 and not induce human podocyte cells apoptosis; however, the downregulation of miR-217 could bring about an opposite change.

**Conclusions:**

miR-217 is a useful diagnostic biomarker and is involved in human podocyte cells apoptosis via targeting TNFSF11 in membranous nephropathy.

## 1. Introduction

Membranous nephropathy (MN) is a renal disease, is a common factor causing nephrotic syndrome in adults, and eventually causes end-stage renal failure for many patients [1–4]. The pathological mechanism of MN is mostly associated with phospholipase A2 receptor antibody; a combination of phospholipase A2 receptor antibody and corresponding antigen on the podocytes forms in the immune complex and then activates the C5b-9 complex through the relative channels, damages the podocytes, destroys the glomerular filtration barrier, and generates proteinuria [5–7]. This disease has been reported to contribute to an immune response to the self-antigens expressed on the podocyte cell and is similar to many immune diseases [8]. Nowadays, there are no specific diagnostic biomarkers in MN.

However, the diagnosis of MN based on clinical characteristics alone is incomplete and usually needs to be confirmed by the histological and immunohistochemical analyses under renal biopsy [9]. In addition, renal biopsy is a kind of trauma for these patients. There is an urgent need to find easy and noninvasive biomarkers to measure and accurately predict long-term outcomes. Therefore, it is very meaningful to find a new diagnostic strategy to provide early and accurate diagnosis for MN.

MicroRNAs are a kind of small noncoding RNAs, which are involved in the posttranscriptional regulation of gene expression by binding the translation section and lead to either mRNA degradation or translational inhibition and have been found to regulate crucial biological processes, including proliferation, differentiation, and apoptosis [10–12]. Some studies have shown a compact link between microRNAs and kidney disease, and many abnormal microRNAs play a significant role in the pathogenesis of kidney disease [13–15]. It has been reported that some microRNAs in the peripheral blood are similar to the microRNAs in tissues [16, 17]; microRNA species are resistant to ribonuclease digestion and are present in serum or plasma [18]. Some studies showed that some microRNAs, including those in the tissue, serum, or plasma, have the ability to diagnose some disorders. For example, circulating miR-221-5p, miR-380-3p, miR-556-5p, miR-758-3p, and miR-3074-3p are potential diagnostic biomarkers of lupus nephritis in patients with systemic lupus erythematosus [13]. miR-320 could be a novel diagnosis and treatment target in renal ischemic reperfusion injury [19]. miR-193b-3p could also be a predictive biomarker in chronic kidney disease undergoing radical nephrectomy for renal cell carcinoma [20].

A previous study showed that there were 20 upregulated and downregulated microRNAs with the highest fold changes in the peripheral blood from patients with MN, and miR-217 was most significantly downregulated, with more than 10-fold reduction in the peripheral blood [21]. The expression of miR-217 in pathological tissues is a useful diagnostic biomarker on MN and its possible pathological mechanism still remains unclear. Therefore, in this study, we investigated the expression of miR-217 in pathological tissues and peripheral blood from MN and whether this microRNA is a diagnostic biomarker for MN. In addition, we also investigated the possible pathological mechanism of this microRNA in MN.

## 2. Patients and Methods

### 2.1. Patient Samples

84 patients with membranous nephropathy (MN), 67 patients with diabetic nephropathy (DN), 82 patients with focal segmental glomerulosclerosis (FSGS), and 72 age- and sex-matched control patients were enrolled in this study at the Sichuan Cancer Hospital, Chengdu, China, between December 2014 and February 2016. Inclusion criteria were age < 65 years, the excretion of urine albumin > 30 mg/24 h, and serum creatinine level < 3 mg/dL. MN was diagnosed according to the previously described criteria [22], FSGS was diagnosed according to the previously described criteria [23], and DN was also diagnosed according to the previously described criteria [24]. Exclusion criteria were secondary FSGS, MN, and DN; other chronic kidney diseases; cancer; or brain, heart, liver, or other systems' diseases. The control patients were enrolled from the same hospital and had no any kidney disease. The study was approved by the Sichuan Cancer Hospital and Human Research Ethics Committee and written informed consent was obtained from all participants. The clinical registration has been completed (researchregistry2622).

### 2.2. Renal Tissue and Plasma Preparation

Pathological renal tissues were collected from patients undergoing renal biopsy and peripheral blood was also collected from these patients. All samples were assigned within 2 hours after collection. The tissue samples were collected in free ribonuclease tubes and stored at −80°C. The blood samples were centrifuged at 3,000*g* for 5 minutes and plasma supernatants were also collected in free ribonuclease tubes to be centrifuged at 15,000*g* for 5 min for eliminating cell debris and stored at −80°C.

### 2.3. Cell Culture

The human podocyte cell line AB8/13 was purchased from American Type Culture Collection and cultured. In brief, human podocyte cells were maintained at 33°C for proliferation and cultured at 37°C to induce differentiation for 14–16 days. An inverted microscope was used to observe the cell morphology. When the human podocyte cells grew to about 80% confluence, they were exposed to 100 nmol/L Angiotensin II (Sigma, Germany) for 24 h.

### 2.4. Transfection of Relevant MicroRNA

Synthetic miR-217 mimics, miR-217 inhibitor, and the relative controls (empty vector) were purchased from BioVectra (Shanghai, China). Synthetic microRNAs were transfected into human podocyte cells to induce upregulation and downregulation of miR-217 with Lipofectamine 2000™ (Invitrogen, MA, USA), according to the manufacturer's protocol. After being infected, the cells were incubated for 8 hours and cultured in a fresh medium for further experiments.

### 2.5. RNA Extraction and qRT-PCR

Total RNA was isolated with the RNeasy Plus Mini Kit (Qiagen), according to the manufacturer's protocols. Total RNA concentrations were detected by the NanoVue plus (GE Healthcare, USA) for RNA concentrations. The cDNA was synthesized with the PrimeScript RT Reagent Kit (TaKaRa, China). The TaqMan miRNA assay (Applied Biosystems, USA) was used for quantification of microRNAs at 95°C for 10 min, followed by 95°C for 15 s (40 cycles) and 60°C for 1 min (40 cycles). The miR-217 was measured with the TaqMan miRNA assays and U6 as an internal control. The expression of miR-217 was evaluated based on the threshold cycle (Ct) as *n* = 2 − ΔΔCt, where ΔCt = Ct related microRNA−Ct U6 and ΔΔCt =  ΔCt experimental−ΔCt control.

### 2.6. The Standard Curves for Absolute Quantification of Plasma miR-217

Total RNA was isolated with the RNeasy Plus Mini Kit (Qiagen), according to the manufacturer's protocols. The synthetic* C. elegans* microRNAs (cel-miR-39, cel-miR-54, and cel-miR-238) were established and validated to normalize for the microRNAs in the plasma. Synthetic single-stranded RNA oligonucleotide miR-217 (miRBase release v.19.0) was purchased from Shanghai GenePharma, China. Synthetic miRNAs were input into the RT reaction according to the range of copies from reference [25, 26]. The standard curves for miR-217 were plotted by Ct values versus copy number of the synthetic miRNAs. Copies of endogenous miRNAs in plasma were then approximated according to their Ct values and the standard curve. A normalization factor was calculated by the Ct values of the three synthetic spiked-in* C. elegans* miRNAs [25, 26].

### 2.7. Prediction of Target Gene

Databases of miRanda, TargetScan, and PicTar were used to predict the potential target genes of miR-217. Among those target genes, the gene of TNFSF11 had a binding site in the 3′UTR of miR-217.

### 2.8. Dual-Luciferase Reporter Assay

Luciferase reporter vector was purchased from Saierbio (Tianjin, China) and the QuickChange Lightning kit (Stratagene, CA, USA) was used for site-directed mutagenesis. Expression miR-217 plasmid and TNFSF11 wild-type and mutated 3′UTR luciferase reporter were cotransfected into HEK293T cells. Renilla luciferase (pRL-TK Vector, Promega, USA) served as the control. The luciferase signal was measured with the Dual-Luciferase Reporter Assay System (Promega, USA).

### 2.9. Western Blot

A lysis buffer was used to extract whole cell lysates for western blotting, and a protein assay kit (Beyotime, China) was used to determine total protein concentration according to the manufacturer's instructions. Total protein (20 *μ*g) was boiled and then chilled and separated and finally transferred to the PVDF membrane (Millipore, USA). The membranes were incubated overnight with primary antibody and incubated with secondary anti-rabbit or anti-mouse horseradish peroxidase-conjugated antibodies according to the manufacturer's protocols (Millipore, USA).

### 2.10. TUNEL Assay

The apoptosis of podocytes was analyzed using TUNEL assay (Roche, USA) following the instructions strictly. In short, cell suspension was attached to the surface of a glass slide with polylysine and fixed with 4% paraformaldehyde. Paraffin sections were used and cells were incubated with terminal deoxynucleotidyl transferase using FITC-labeled nucleotides. The percentage of TUNEL-positive cells was calculated as follows: TUNEL-positive cells/total number of cells × 100%.

### 2.11. Statistical Analysis

The data were presented as mean ± SD, median (range), or categorical data. The unpaired *t*-test and the one-way analysis of variance test were used to analyze continuous data. The chi-square test was used to analyze categorical data. Receiver operating characteristic (ROC) curve analysis was used for the separation level. A *p* value < 0.05 was considered to represent a statistically significant difference.

## 3. Results

### 3.1. The Relative Expression of miR-217 in Tissue and Plasma of Membranous Nephropathy

**Figure 1:**
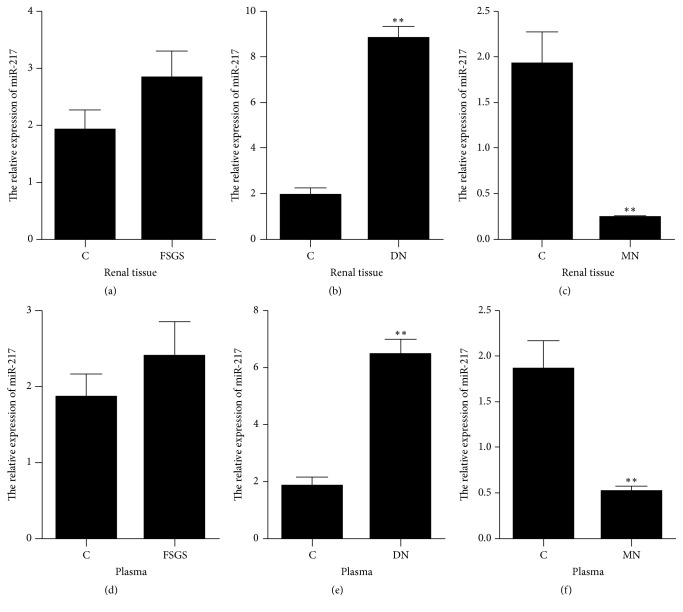
The relative expression of miR-217 in tissue and plasma from different patients. C: control patients (*n* = 72); FSGS: focal segmental glomerulosclerosis patients (*n* = 82); DN: diabetic nephropathy patients (*n* = 67); MN: membranous nephropathy patients (*n* = 84); ^*∗∗*^*p* value < 0.01.

Renal tissue was collected from those patients undergoing renal biopsy and plasma was isolated from the blood sample. The relative expression of miR-217 was detected by the qRT-PCR method. The results showed that, compared with control patients, the expression of miR-217 was not statistically different in renal tissue and plasma from FSGS, was significantly upregulated in those from the DN (fold change = 4.27 in renal tissue, fold change = 3.25 in plasma), and was significantly downregulated in those from MN (fold change = −6.95 in renal tissue, fold change = −4.12 in plasma) (Figures 1(a)–1(f)).

### 3.2. The Absolute Expression of miR-217 in Plasma of Membranous Nephropathy

**Figure 2:**
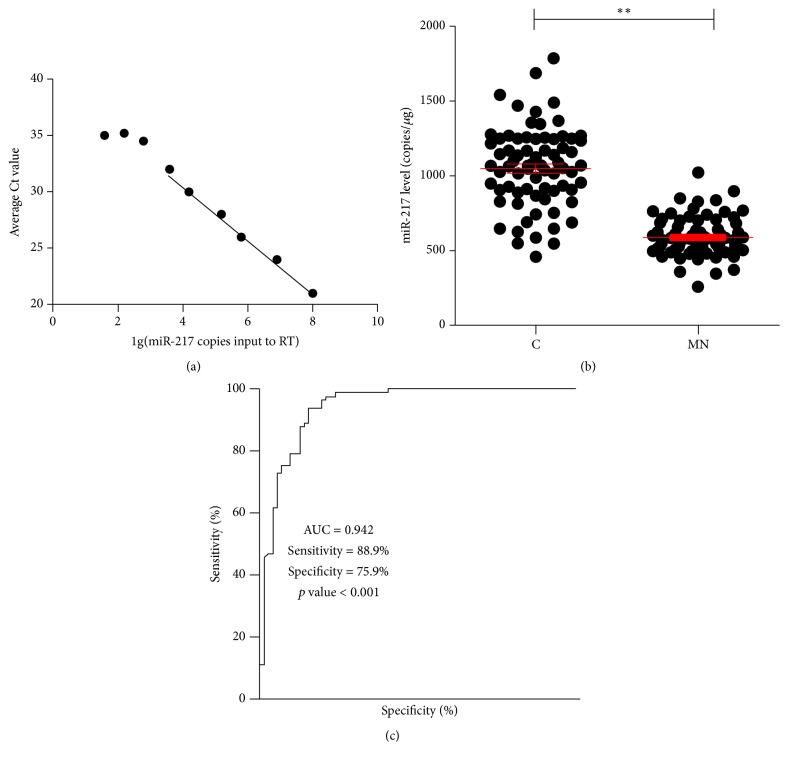
The absolute expression of plasma miR-217 and receiver operating characteristic (ROC) curve analysis. C: control patients (*n* = 72); MN: membranous nephropathy patients (*n* = 84); ^*∗∗*^*p* value < 0.01.

The standard curves of miR-217 were analyzed by Ct values and copy number of the synthetic microRNAs (Figure 2(a)). The absolute expression of miR-217 was detected by the qRT-PCR method. The results showed that, compared with control patients, the absolute expression of plasma miR-217 was also significantly downregulated in MN* (fold change = −2.02 in plasma)* (Figure 2(b)).

### 3.3. Receiver Operating Characteristic (ROC) Curve Analysis between MN and Healthy Controls from Plasma miR-217

The absolute expression of plasma miR-217 was enrolled in the ROC curve analysis. The results showed that plasma miR-217 reflected obvious separation between MN (*n* = 84) and control patients (*n* = 72), with an AUC of 0.942 (95% confidence interval = 0.904 to 0.979), a cutoff value < 750.0 copies/ul, and sensitivity and specificity of 88.9% and 75.9% (Figure 2(c)).

### 3.4. TNFSF11 Was the Target Gene of miR-217

**Figure 3:**
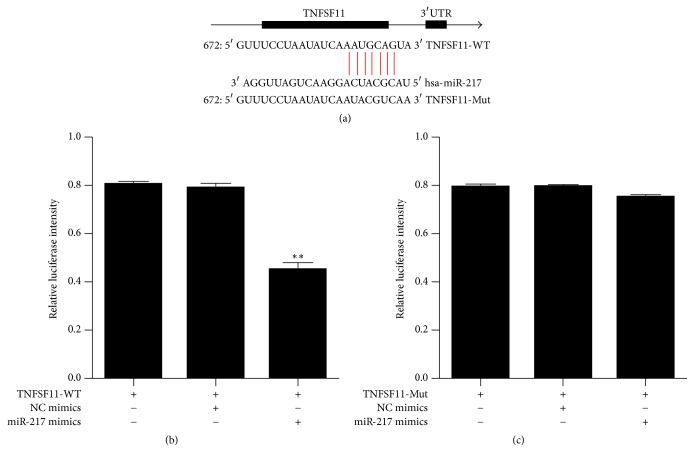
The dual-luciferase reporter assay of miR-217. (a) Putative has-miR-217 binding sequence in the TNFSF11 3′UTR and the site-directed mutant TNFSF11 3′UTR. (b, c) The WT or Mut reporter plasmids or NC or miR-217 mimics were cotransfected into HEK293T cells; ^*∗∗*^*p* value < 0.01.

According to target gene prediction analysis, TNFSF11 had a putative binding site in the 3′UTR of miR-217 and might be the target gene of miR-217 (Figure 3(a)). The dual-luciferase reporter assay method was used to further investigate whether miR-217 directly targeted TNFSF11. The results showed that miR-217 inhibited luciferase activity under 3′UTR of wild-type (WT) TNFSF11 (Figure 3(b)). In addition, miR-217 did not inhibit luciferase activity under 3′UTR of mutated (Mut) TNFSF11 (Figure 3(c)).

### 3.5. The Expression of TNFSF11 in Membranous Nephropathy

**Figure 4:**
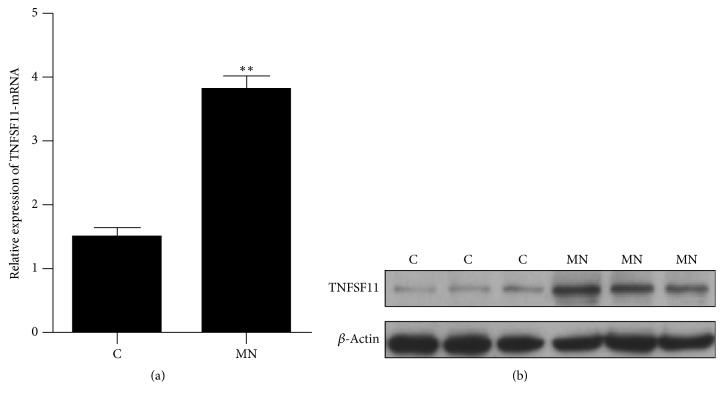
The expression of TNFSF11 in membranous nephropathy. (a) The gene expression of TNFSF11; (b) the protein expression of TNFSF11. C: random control patients; MN: random membranous nephropathy patients; ^*∗∗*^*p* value < 0.01.

Renal tissue was collected from those patients undergoing renal biopsy and the expression of TNFSF11 was detected by qRT-PCR and western blot method. The results showed that, compared with control patients, the gene and protein expression of TNFSF11 was significantly upregulated in MN (Figure 4).

### 3.6. The Effects of miR-217 on the Apoptosis in Human Podocyte Cells

**Figure 5:**
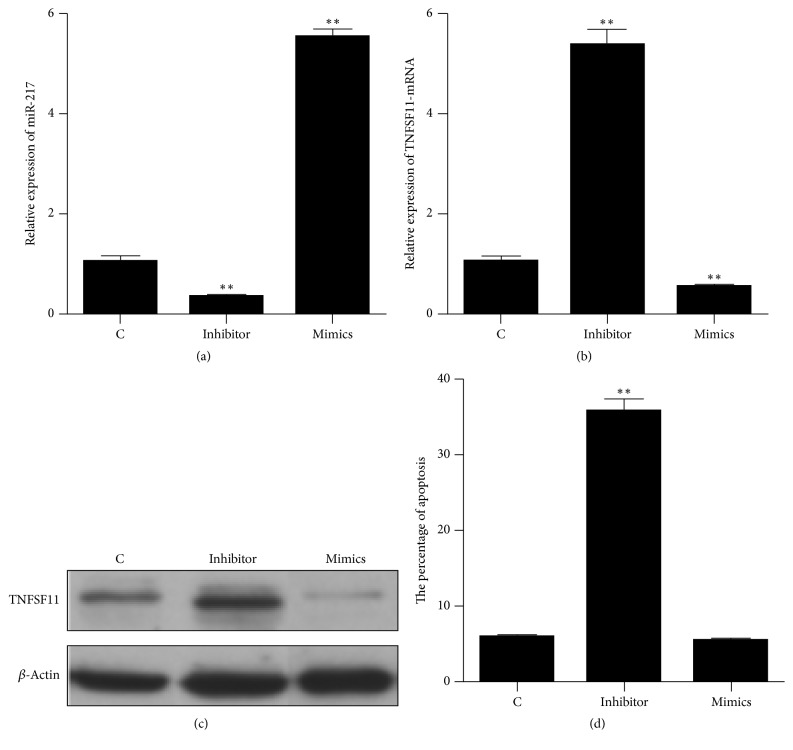
The effects of miR-217 on the apoptosis in human podocyte cells. (a) The gene expression of miR-217; (b) the gene expression of TNFSF11; (c) the protein expression of TNFSF11. C: control (podocyte cells infected with empty vector). Inhibitor: podocyte cells infected with miR-217 inhibitor. Mimics: podocyte cells infected with miR-217 mimics. ^*∗∗*^*p* value < 0.01, compared with control.

Synthetic miR-217 mimics, miR-217 inhibitor, and the relative controls were transfected into human podocyte cells. The expression of miR-217 and TNFSF11 was detected by qRT-PCR and western blot method, and the TUNEL assay was used to evaluate cell apoptosis. The results showed that the expression of miR-217 was significantly downregulated and TNFSF11 was significantly upregulated with miR-217 inhibitor, and miR-217 was significantly upregulated and TNFSF11 was significantly downregulated with miR-217 mimics (Figures 5(a), 5(b), and 5(c)). The upregulation of miR-217 could not induce human podocyte cells apoptosis; however, the downregulation of miR-217 could induce human podocyte cells apoptosis (Figure 5(d)).

## 4. Discussion

Membranous nephropathy (MN) is a common nephrotic syndrome in adults and leads to severe complications [27]. Nowadays, histological and immunohistochemical analyses under renal biopsy are considered as the gold diagnostic standard of this disease [9], but this method is traumatic. Although this method is considered as safe progress with low complications, a noninvasive diagnostic method may be preferable. Some studies demonstrated that circulating microRNAs were useful diagnostic biomarkers in various diseases. However, the question of whether circulating microRNAs can be diagnostic biomarkers for membranous nephropathy (MN) still remains unclear. Therefore, the new diagnostic strategy is very important for this disease. In this study, for the first time, we demonstrated that absolute quantification of plasma miR-217 was able to be a useful diagnostic biomarker in MN, and we discussed the possible mechanism of miR-217 in MN and provided a new diagnostic strategy for MN.

It has been reported that microRNAs are small, noncoding RNAs and have the ability to regulate protein-coding genes by binding the translation section and lead to either mRNA degradation or translational inhibition [28]. Recently, microRNAs in tissues have also been reported to be released in the peripheral blood, and circulating microRNAs can be associated with a specific pathophysiological state [29]. Whether miR-217 in tissue was released in the peripheral blood in MN remains unclear. In this study, firstly, we showed that the expression of miR-217 was consistent in the pathological renal tissues and plasma in MN; abnormal expression of renal tissue miR-217 possibly contributed to abnormal expression of plasma miR-217 (relative expression of fold change = −6.95 in renal tissue, relative expression of fold change = −4.12 in plasma, and absolute expression of fold change = −2.02 in plasma). For microRNAs, relative expression of microRNA is qualitative, while absolute expression of microRNA is quantitative; due to the difference of experimental methods, the fold change of detected microRNAs was different. In addition, due to the samples, the instruments, and so forth, relative expression of the detected microRNA was not an accurate value, and it is not accurate to say that the relative quantification of microRNAs was used as a diagnostic biomarker in MN. Therefore, in this study, we detected the absolute quantification of plasma miR-217. The copies of microRNAs in each sample had been reported to be multiplied by the normalization factor corresponding to the sample to obtain a normalized copy number [25]. The results showed that, compared with control patients, the absolute expression of plasma miR-217 was also significantly downregulated in MN (fold change = −2.02 in plasma). According to the absolute expression of plasma miR-217, plasma miR-217 reflected obvious separation between MN and control patients, with an AUC of 0.942 (95% confidence interval = 0.904 to 0.979), a cutoff value < 750 copies/ul, and sensitivity and specificity of 88.9% and 75.9%. Therefore, plasma miR-217 was a useful diagnostic biomarker in MN.

To date, the recognized pathological mechanism of MN is the combination of relative phospholipase A2 antibody and receptor that damages the podocytes and then destroys the glomerular filtration barrier and generates proteinuria [5–7]. Some studies have reported that TNF-*α* could induce cytosolic phospholipase A2 expression via JNK1/2- and p38 MAPK-dependent AP-1 activation [30]; TNF-*α* could also induce cytosolic phospholipase A2 expression via inhibition of PKC*α*-dependent NADPH oxidase/ROS and NF-*κ*B [31]. Therefore, the tumor necrosis factor (TNF) cytokine could induce the expression of phospholipase A2. As regards the tumor necrosis factor superfamily member 11 (TNFSF11), this gene encodes a member of the tumor necrosis factor (TNF) cytokine family, which is shown to be the cell survival factor and is involved in the regulation of T-cell-dependent immune response [32]. In MN, overexpression of TNFSF11 increases the tumor necrosis factor (TNF) cytokine, induces the expression of phospholipase A2, and is involved in the progress of MN. In addition, this protein is shown to activate antiapoptotic kinase AKT/PKB through a signaling complex involving SRC kinase and tumor necrosis factor receptor-associated factor (TRAF) 6, which indicated that this protein may play a role in the regulation of cell apoptosis [33, 34]. Some studies showed that TNF and TNF receptor superfamilies were involved in the pathogenesis of podocyte injury and apoptosis [35, 36]. The reduced number of podocytes is a critical part in the pathological mechanism of MN [37]. In humans, microRNAs are reported to regulate >60% of coding genes and have been involved in various biological processes, including cell proliferation, cell apoptosis, developmental patterning, and organ development [38, 39]. Whether miR-217 regulated the expression of TNFSF11 and whether this relation between miR-217 and TNFSF11 contributed to the pathological mechanism of MN remain unclear. In this study, the dual-luciferase reporter assay confirmed that TNFSF11 was the target gene of miR-217. In addition, compared with control patients, the gene and protein expression of TNFSF11 was significantly upregulated in MN. Therefore, miR-217 possibly contributed to the pathological mechanism of MN via targeting TNFSF11. In order to verify this hypothesis, synthetic miR-217 mimics and miR-217 inhibitor were transfected into human podocyte cells. The results showed that the miR-217 regulated TNFSF11 in human podocyte cells; the upregulation of miR-217 could not induce human podocyte cells apoptosis; however, the downregulation of miR-217 could induce human podocyte cells apoptosis. Therefore, miR-217 is involved in human podocyte cells apoptosis via targeting TNFSF11 and contributed to the pathological mechanism of MN.

The study also had some limitations. First, the sample size was small. Second, all of the patients only had membranous nephropathy, diabetic nephropathy, or focal segmental glomerulosclerosis, not other relative diseases.

In summary, our study identifies that miR-217 is a useful diagnostic biomarker and is involved in human podocyte cells apoptosis via targeting TNFSF11 in membranous nephropathy and provides a new diagnostic strategy for membranous nephropathy.

## References

[1] Aaltonen S., Honkanen E. (2011). Outcome of idiopathic membranous nephropathy using targeted stepwise immunosuppressive treatment strategy. *Nephrology Dialysis Transplantation *.

[2] Ponticelli C., Passerini P. (2010). Can prognostic factors assist therapeutic decisions in idiopathic membranous nephropathy?. *Journal of Nephrology*.

[3] Filippone E. J., Farber J. L. (2016). Membranous nephropathy in the kidney allograft. *Clinical Transplantation*.

[4] Cattran D. C., Kim E. D., Reich H., Hladunewich M., Kim S. J. (2017). Membranous nephropathy: quantifying remission duration on outcome. *Journal of the American Society of Nephrology*.

[5] Nangaku M., Shankland S. J., Couser W. G. (2005). Cellular response to injury in membranous nephropathy. *Journal of the American Society of Nephrology*.

[6] Lee S. J., Borsting E., Declèves A.-E., Singh P., Cunard R. (2012). Podocytes express IL-6 and lipocalin 2/neutrophil gelatinase-associated lipocalin in lipopolysaccharide-induced acute glomerular injury. *Nephron Experimental Nephrology*.

[7] Tomas N. M., Hoxha E., Reinicke A. T. (2016). Autoantibodies against thrombospondin type 1 domain-containing 7A induce membranous nephropathy. *The Journal of Clinical Investigation*.

[8] Couser W. G. (2005). Membranous nephropathy: A long road but well traveled. *Journal of the American Society of Nephrology*.

[9] Praga M., Rojas-Rivera J. (2012). Glomerular disease: Predicting outcomes in idiopathic membranous nephropathy. *Nature Reviews Nephrology*.

[10] Bartel D. P. (2004). MicroRNAs: genomics, biogenesis, mechanism, and function. *Cell*.

[11] Kim V. N., Nam J. W. (2006). Genomics of microRNA. *Trends in Genetics*.

[12] Krol J., Loedige I., Filipowicz W. (2010). The widespread regulation of microRNA biogenesis, function and decay. *Nature Reviews Genetics*.

[13] Navarro-Quiroz E., Pacheco-Lugo L., Lorenzi H. (2016). High-throughput sequencing reveals circulating miRNAs as potential biomarkers of kidney damage in patients with systemic lupus erythematosus. *PLoS ONE*.

[14] Fan P., Chen C., Chen Y., Chang Y., Chu P. (2016). MicroRNAs in acute kidney injury. *Human Genomics*.

[15] Hajarnis S., Lakhia R., Patel V., Li X. (2015). MicroRNAs and Polycystic Kidney Disease. *Polycystic Kidney Disease*.

[16] Wang J. L., Wang X., Yang D., Shi W. (2016). The Expression of MicroRNA-155 in plasma and tissue is matched in human laryngeal squamous cell carcinoma. *Yonsei Medical Journal*.

[17] Jiao Y., Zhu M., Mao X., Long M., Du X., Wu Y. (2015). MicroRNA-130a expression is decreased in Xinjiang Uygur patients with type 2 diabetes mellitus. *American Journal of Translational Research*.

[18] Chen X., Ba Y., Ma L. (2008). Characterization of microRNAs in serum: a novel class of biomarkers for diagnosis of cancer and other diseases. *Cell Research*.

[19] Guclu A., Kocak C., Kocak FE., Akcilar R., Dodurga Y., Akcilar A. (2016). Micro RNA-320 as a novel potential biomarker in renal ischemia reperfusion. *Renal Failure*.

[20] Trevisani F., Ghidini M., Larcher A. (2016). MicroRNA 193b-3p as a predictive biomarker of chronic kidney disease in patients undergoing radical nephrectomy for renal cell carcinoma. *British Journal of Cancer*.

[21] Chen W., Lin X., Huang J. (2014). Integrated profiling of microRNA expression in membranous nephropathy using high-throughput sequencing technology. *International Journal of Molecular Medicine*.

[22] Qin W., Beck L. H., Zeng C. (2011). Anti-phospholipase A2 receptor antibody in membranous nephropathy. *Journal of the American Society of Nephrology*.

[23] Zhang Q., Zeng C., Cheng Z., Xie K., Zhang J., Liu Z. (2012). Primary focal segmental glomerulosclerosis in nephrotic patients: Common complications and risk factors. *Journal of Nephrology*.

[24] Alberti K. G. M. M., Zimmet P. Z. (1998). Definition, diagnosis and classification of diabetes mellitus and its complications. Part 1: diagnosis and classification of diabetes mellitus. Provisional report of a WHO consultation. *Diabetic Medicine*.

[25] Mitchell P. S., Parkin R. K., Kroh E. M. (2008). Circulating microRNAs as stable blood-based markers for cancer detection. *Proceedings of the National Acadamy of Sciences of the United States of America*.

[26] Kroh E. M., Parkin R. K., Mitchell P. S., Tewari M. (2010). Analysis of circulating microRNA biomarkers in plasma and serum using quantitative reverse transcription-PCR (qRT-PCR). *Methods*.

[27] Idasiak-Piechocka I., Oko A., Łochyńska-Bielecka K., Skrobańska B. (2009). Efficacy and safety of low-dose chlorambucil in nephrotic patients with idiopathic membranous nephropathy. *Kidney and Blood Pressure Research*.

[28] Flynt A. S., Lai E. C. (2008). Biological principles of microRNA-mediated regulation: shared themes amid diversity. *Nature Reviews Genetics*.

[29] Cortez M. A., Calin G. A. (2009). MicroRNA identification in plasma and serum: a new tool to diagnose and monitor diseases. *Expert Opinion on Biological Therapy*.

[30] Lee I.-T., Lin C.-C., Cheng S.-E., Hsiao L.-D., Hsiao Y.-C., Yang C.-M. (2013). TNF-*α* Induces Cytosolic Phospholipase A2 Expression in Human Lung Epithelial Cells via JNK1/2- and p38 MAPK-Dependent AP-1 Activation. *PLoS ONE*.

[31] Chi P.-L., Liu C.-J., Lee I.-T., Chen Y.-W., Hsiao L.-D., Yang C.-M. (2014). HO-1 induction by CO-RM2 attenuates TNF- *α* -induced cytosolic phospholipase A2 expression via inhibition of PKC *α* -dependent NADPH oxidase/ROS and NF- B. *Mediators of Inflammation*.

[32] Anderson D. M., Maraskovsky E., Billingsley W. L. (1997). A homologue of the TNF receptor and its ligand enhance T-cell growth and dendritic-cell function. *Nature*.

[33] Yi T., Lee H.-L., Cha J.-H. (2008). Epidermal growth factor receptor regulates osteoclast differentiation and survival through cross-talking with RANK signaling. *Journal of Cellular Physiology*.

[34] Sugatani T., Alvarez U. M., Hruska K. A. (2003). Activin A stimulates I*κ*B-*α*/NF*κ*B and RANK expression for osteoclast differentiation, but not AKT survival pathway in osteoclast precursors. *Journal of Cellular Biochemistry*.

[35] Saito Y., Okamura M., Nakajima S. (2010). Suppression of nephrin expression by TNF-*α* via interfering with the cAMP-retinoic acid receptor pathway. *American Journal of Physiology-Renal Physiology*.

[36] Sanchez-Nio M. D., Benito-Martin A., Gonalves S. (2010). TNF superfamily: a growing saga of kidney injury modulators. *Mediators of Inflammation*.

[37] Ronco P., Debiec H. (2006). New insights into the pathogenesis of membranous glomerulonephritis. *Current Opinion in Nephrology and Hypertension*.

[38] Esquela-Kerscher A., Slack F. J. (2006). Oncomirs—microRNAs with a role in cancer. *Nature Reviews Cancer*.

[39] Skalsky R. L., Cullen B. R. (2010). Viruses, microRNAs, and host interactions. *Annual Review of Microbiology*.

